# Improving the Production of L-Phenylalanine by Identifying Key Enzymes Through Multi-Enzyme Reaction System *in Vitro*

**DOI:** 10.1038/srep32208

**Published:** 2016-08-25

**Authors:** Dongqin Ding, Yongfei Liu, Yiran Xu, Ping Zheng, Haixing Li, Dawei Zhang, Jibin Sun

**Affiliations:** 1 Tianjin Institute of Industrial Biotechnology, Chinese Academy of Sciences, Tianjin 300308, People’s Republic of China; 2 Department of Food Science and Engineering, School of Food, Nanchang University, Nanchang 330029, People’s Republic of China; 3 Key Laboratory of Systems Microbial Biotechnology, Chinese Academy of Sciences, Tianjin 300308, People’s Republic of China.

## Abstract

L-Phenylalanine (L-Phe) is an important amino acid used in both food and medicinal applications. We developed an *in vitro* system that allowed a direct, quantitative investigation of phenylalanine biosynthesis in *E. coli*. Here, the absolute concentrations of six enzymes (AroK, AroL, AroA, AroC, PheA and TyrB) involved in the shikimate (SHIK) pathway were determined by a quantitative proteomics approach and *in vitro* enzyme titration experiments. The reconstitution of an *in vitro* reaction system for these six enzymes was established and their effects on the phenylalanine production were tested. The results showed that the yield of phenylalanine increased 3.0 and 2.1 times when the concentrations of shikimate kinase (AroL) and 5-enolpyruvoyl shikimate 3-phosphate (EPSP) synthase (AroA) were increased 2.5 times. Consistent results were obtained from *in vivo* via the overexpression of AroA in a phenylalanine-producing strain, and the titer of phenylalanine reached 62.47 g/l after 48 h cultivation in a 5-liter jar fermentor. Our quantitative findings provide a practical method to detect the potential bottleneck in a specific metabolic pathway to determine which gene products should be targeted to improve the yield of the desired product.

L-Phe is an essential amino acid for humans and most livestock. It is used in feed, food additives, taste and aroma enhancers, pharmaceuticals or as building blocks for drugs, dietary supplements, nutraceuticals, and ingredients in cosmetics^1^. Notably, L-Phe is used in the production the sweetener aspartame, which has a steadily increasing world-wide demand^2^. Currently, L-Phe is obtained by chemical, enzymatic or microbial processes. In recent years, there has been increased interest in producing L-Phe through microbial fermentation, especially by metabolically engineered strains of *E. coli*, which have a high growth rate and well-defined physiological characteristics^3^.

L-Phe biosynthesis and its regulation have been extensively investigated in *E. coli.* The shikimate pathway serves as the primary source of production of L-Phe. Two shikimate kinases, AroK and AroL, catalyze the formation of shikimate 3-phosphate (S3P) from shikimate and ATP. The expression of *aroL* is regulated by TyrR with tyrosine or tryptophan as a co-repressor^4^,^5^. In contrast to AroL, the activity of AroK in the cell is independent of both the amount of extracellular aromatic amino acids and the level of *tyrR* gene product^6^,^7^. 5-Enolpyruvyl shikimate 3-phosphate synthase (AroA) catalyzes the transfer of the enolpyruvoyl moiety from phosphoenolpyruvate (PEP) to the hydroxyl group of carbon 5 of S3P with the elimination of phosphate to produce EPSP^8^,^9^,^10^,^11^. This is an addition-elimination reaction, which introduces the three-carbon fragment destined to become the side chain of phenylalanine. The branch point compound that serves as the starting substrate for the three terminal pathways of aromatic amino acid biosynthesis, chorismic acid (CHA), is obtained through the conversion of EPSP to chorismate catalyzed by chorismate synthetase (AroC)^12^,^13^. This enzyme catalyzes the phosphate elimination by a 1,4-elimination mechanism that proceeds with anti-stereochemistry^14^. Additionally, this enzyme is oxygen sensitive and may remain inactive under aerobic conditions^15^, but it can be activated by a reduced flavin adenine dinucleotide (FAD)-regenerating system in an atmosphere of H_2_. This was accomplished by the reduction of FAD with reduced nicotinamide adenine dinucleotide (NADH) and mammalian diaphorase or bacterial NADH dehydrogenase^16^. Bifunctional chorismate mutase / prephenate dehydratase (PheA) carries out the second step in phenylalanine biosynthesis as well as in the parallel biosynthetic pathways for the production of the aromatic amino acids tyrosine and phenylalanine. The native enzyme is a dimer of identical subunits each containing a dehydratase active site, a mutase active site and a phenylalanine binding site^17^,^18^. L-Phe was shown to feedback-inhibit both the chorismate mutase and prephenate dehydratase activities of the enzyme by an allosteric mechanism^6^. Finally, a tyrosine-repressible aromatic amino acid aminotransferase (TyrB) catalyzes the transamination of L-Glutamic acid and phenylpyruvate to yield L-Phe^19^,^20^,^21^.

To some extent, it is possible to enhance the specific productivity of L-Phe in *E. coli* by manipulating known catalytic enzymes. Due to the complexity and interconnectivity of metabolic networks, the creation of a highly efficient cell factory for the production of a target protein often requires multiple alterations, including overexpressing key enzymes, deleting genes involved in off-pathway processes or that lead to byproducts, increasing the expression level of efflux proteins for the removal of feedback inhibition, or introducing external enzymes with high activity^22^,^23^,^24^,^25^,^26^. For instance, Backman *et al.* engineered *E. coli* for L-Phe production, based on overexpressing the *aroF*^WT^ and *pheA*^fbr^ genes and developed an efficient fermentation process within 36 h, with a final L-Phe titer of 50 g/l^27^, and Marjan *et al.* applied direct reduction of the carbon flow to acetate by knocking out *ackA-pta* and *poxB*^28^. Although there are a number of studies on the relationships between the flux of a metabolic system *in vivo* and the absolute concentrations of particular enzymes, the measurement of precise concentrations and activities of the enzymes in the cells are quite challenging. Nevertheless, *in vitro* enzyme reaction system has been well applied in recent years to achieve the guide of engineering the strains^29^. Moreover, label-free proteomics tools will provide an opportunity for the measurement of absolute protein concentrations inside the cells, which will be a new application in the field of metabolic engineering.

Collectively, we took all of these advantages and developed a much easier method to analyze L-phe biosynthesis pathway quantitatively. In this study, the absolute quantities of proteins in the phenylalanine production strain *HD-1* were determined using label-free proteomics. In addition, to establish a multi-enzyme reaction system, we expressed and purified six enzymes from the shikimate pathway (AroK, AroL, AroA, AroC, PheA, and TyrB) and verified their activities. We then reconstituted the shikimate pathway reaction system *in vitro* base on the absolute concentrations of shikimate enzymes obtained from the proteomics data. We found that AroL and AroA were bottlenecks for increasing phenylalanine synthesis flux. We tested overexpression of the *aroL* and *aroA* genes in the production strain and found that overexpression of *aroA* improved the yield of L-Phe by 45.18%. The results showed that the *in vitro* multi-enzyme system was a rational strategy for reconstitution of the shikimate acid pathway components *in vivo* that regulate the production of L-Phe.

## Materials and Methods

### Plasmid, bacterial strains and medium

Plasmid pET28a was reserved in our lab. The host bacterial DH5α and BL21(DE3) were purchased from invitrogen company. The *E. coli HD-1* strain for phenylalanine production was derived from *E.coli* W3110 strain that initially undergone multiple random mutagenesis for the enhanced production of L-phenylalanine and then stepwise rational metabolic engineering. The genome sequences of the *HD-1* strain were determined and compared (Table 1 and Supplementary Tables 2–3) with our knowledge. All the bacteria are kept in 30% of the glycerin and stored in −80 °C before used.

LB medium (per liter): tryptone 10 g, yeast extract 5 g, NaCl 10 g. Seed medium (per liter): glucose 20 g, (NH4)_2_SO_4_ 10 g, KH_2_PO_4_ 1.5 g, MgSO_4_ 5 g, yeast extract 4 g, FeSO_4_.7 H_2_O 15 mg, sodium citrate 0.5 g and VB1 100 mg. Fermentation medium (per liter): glucose 20 g, (NH4)_2_SO_4_ 10 g, KH_2_PO_4_ 5 g, MgSO_4_ 5 g, yeast extract 4 g, FeSO_4_.7 H_2_O 15 mg, MnSO_4_.H_2_O 15 mg and Betaine monohydrate 1 g.

### Material, enzymes and chemicals

The substrates SHIK, phenylpyruvic acid, ATP, KCl, MgCl_2_, PEP, FAD, NADH, β-Mercaptoethanol, L-Glutamic acid, pyridoxal phosphate were purchased from Sigma (St. Louis, MO, USA). Prime Star Taq polymerase was purchased from Takara and the restriction enzymes and T4 DNA ligase were purchased from NEB. Ni-NTA agarose resin was supplied by GE Healthcare for His-tagged protein purification. Na_2_HPO_4_ and other organic solvents used for HPLC were purchased from Merck (German). Other chemicals used in this article otherwise demonstrated were purchased from Solarbio (Beijing, China).

All kits and markers used for the construction of clones were from Omega Bio-tek, Inc (USA).

### Plasmid Construction

The genomic DNA of the phenylalanine producing *E. coli* strain *HD-1* was used as the template for amplification of the phenylalanine synthesis genes. All enzymes genes were amplified by PCR using primers as described in the Supplementary Table 1. After amplification, the DNA fragments were purified from agarose gel, and subsequently inserted into the pET28a vector using restriction enzymes and T4 DNA ligase. The plasmids and strains used in this study are shown in Table 1.

### Enzyme expression and purification

To purify the enzymes, the corresponding plasmids were introduced into *E. coli* BL21 (DE3). The DE3 cells were grown in 100 ml of LB broth at 37 °C and induced by adding 0.4 mM IPTG when the absorbance at 600 nm reached 0.6. The cells were further grown at 16 °C for 12 h before being harvested. The cell pellet was washed three times with enzyme assay buffer (136.8 mM NaCl, 2.68 mM KCl, 10 mM Na_2_HPO_4_∙12H_2_O, 1.98 mM KH_2_PO_4_), and resuspended in binding buffer (26 mM NaH_2_PO_4_, pH 7.4, 2 M NaCl, 30 mM imidazole, 10 mM β-mercaptoethanol, 10 μM Triton-X100) containing 1% cocktail (protease inhibitors), and then it was disrupted by sonication. After sonication, the cell lysate was centrifuged at 10,000 × g for 30 min at 4 °C and filtered (0.22 μm) to remove cell debris. The supernatant fraction was subjected to Ni2^+^-chelating affinity chromatography containing Ni-NTA agarose equilibrated with H_2_O at 4 °C. The column was washed with binding buffer and then with washing buffer (26 mM NaH_2_PO_4_, 2 M NaCl, 100 mM imidazole, pH 7.4). Bound proteins were then eluted with elution buffer (26 mM NaH_2_PO_4_, 0.5 M NaCl, 500 mM imidazole, pH 7.4). The recombinant protein-containing fractions were dialyzed against storage buffer (100 mM Tris-HCl, 5 mM β-mercaptoethanol) in order to remove the imidazole. After purification, the purified enzymes concentrations were determined using the BCA kit. The purified proteins were stored at −80 °C until use.

### Fermentation of phenylalanine production strains in a 5L-bioreactor

A crude enzyme extraction was prepared from the high phenylalanine-producing strain *HD-1*. After growing 7 hours in a shaking flask of LB, the cells were then inoculated into a 3.5 L fermentation medium in a 5L bioreactor. During the entire fermentation process, the pH was kept at 7.0 with the addition of 25% ammonia water and the temperature was set at 33 °C until the OD_600_ reached 35 (mid log phase) and then was up-shifted to 38 °C to induce the expression of enzymes responsible for L-Phe production^1^. The dissolved oxygen (DO) level was upheld near 40% by adjusting the agitation speed (300–900 rpm) and the aeration rate (2–3 vvm). The glucose concentration was maintained between 0–5 g/l by feeding 800 g/l glucose. The fermentation broth was sampled every two hours to detect phenylalanine concentrations, cell density analysis, and glucose consumption. A sample collected at 32 h was used for the crude enzyme extraction experiment. The cells were harvested by centrifugation for 15 min at 10,000 g at 4 °C. For the crude enzyme extract preparation, the cell pellet was washed three times with enzyme assay buffer. Next, the cells were resuspended in lysis buffer (binding buffer containing 1% cocktail). The crude extracts were obtained by sonication of the cell suspension. Cellular debris was removed by centrifugation (30 min, 10,000 g, 4 °C) and the supernatant was dialyzed at 4 °C. The final crude enzyme extracts were stored at −80 °C.

### Proteomics analysis

The cells for the proteomics analysis were the same as those used for preparation of the cell crude extract. The cells were harvested by centrifugation at 4000 × g for 10 min at 4 °C (Eppendorf 5810R, Germany) and resuspended in lysis buffer (Tris-HCl pH 7.6 0.1 M, DTT 0.1 M, cocktail slice/10 mL, obtained from Calbiotech, Inc, Spring Valley, USA). After the cells were broken by sonication cracking on ice, the supernatant was centrifuged at 4000 × g for 10 min at 4 °C. The supernatant was then transferred to a new tube and proteins were quantified using the BCA Kit. For digestion, the protein pellet from previous step was washed with UA buffer [8M Urea dissolved in 0.1 M Tris-HCl (pH 8.5)] and dissolved in digestion buffer [100 mM TEAB (triethylammonium bicarbonate)] to a final concentration of 1 mg/mL. Equal aliquots were then digested with trypsin (Promega, Madison, USA) overnight at 37 °C.

A NanoLC system (NanoLC 2D Ultra, Eksigent, Massachusettes, USA) equipped with a Triple TOF 5600 mass spectrometer (AB SCIEX, Massachusettes, USA) was used for analysis. Peptides were trapped on a NanoLC pre-column (Chromxp C18-LC-3 μm, size 0.35 × 0.5 mm, Eksigent) and then eluted onto an analytical column (C18-CL-120, size 0.075 × 150 mm, Eksigent) and separated by a 120 min gradient from 5 to 35% Buffer B (Buffer A: 2% ACN, 98% H_2_O, Buffer B: 98% ACN, 2% H2O, 0.1% FA) at a flow rate of 300 nL/min. Full-scan MS was performed in positive ion mode with nano-ion spray voltage of 2.5 kv from 350 to 1500 (m/z), with up to 30 precursors selected for MS/MS (m/z 100–1500) if exceeding a threshold of 125 counts per second (counts/s). Peptides with +2 to +5 charge states were selected for MS/MS. The collision energy (CE) for collision-induced dissociation (CID) was automatically controlled using an Information-Dependent Acquisition (IDA) CE parameter script to achieve optimum fragmentation efficiency. Finally, the relative proportions of the enzymes in the cell could be obtained by comparing the amount of the peptides with the protein molecular masses after the proteomic analysis^30^.

### Determination of phenylpyruvic acid

For the determination of phenylpyruvic acid, 500 μl reaction mixtures were treated with 50 μl of 1 M HCl, and extracted with 5 ml of ether and 1 min vortexing. The ether layer was transferred to a tube containing 3 ml of 2 M NaOH, and mixed for 30 s. After removing the ether layer, the remaining liquid was transferred to an ELISA plate and the absorption of the phenylpyruvate enolate anion was measured at 320 nm. A standard curve was obtained by treating phenylpyruvic acid (0.02 to 0.5 μM) in the same manner.

### Assay of enzyme activities

The activity of the purified shikimate kinase was determined by coupling the release of ADP from the shikimate kinase-catalyzed reaction to the oxidation of NADH using pyruvate kinase (EC 2.7.1.40) and lactate dehydrogenase (EC 1.1.1.27) as coupling enzymes^31^. Shikimate-dependent oxidation of NADH was monitored by the change in absorbance at *A*_340_ (ε = 6,200 M^−1^cm^−1^) at 25 °C. The 500 μl of mixture contained 100 mM Tris-HCl-KOH buffer (pH 7.5), 50 mM KCl, 5 mM MgCl_2_, 1.6 mM shikimic acid, 2.5 mM ATP, 1 mM phosphoenolpyruvate, 0.1 mM NADH, 2.5 U of pyruvate kinase, and 2.7 U of lactate dehydrogenase, and 0.1 to 10 μg of the enzyme being analyzed. A standard curve was obtained by treating NADH (0.005 to 0.15 mM) in the same way.

The activities of the purified enzyme were assayed in two steps. The first part detects phenylpyruvic acid after the first four enzymes were added. The incubation mixture, in a 3 ml anaerobic bottle, contained 100 mM Tris-HCl pH 7.5, SHIK (8 mM), ATP (8 mM), KCl (50 mM), MgCl_2_ (5 mM), PEP (4 mM), FAD (1 mM), NADH (5 mM), β-mercaptoethanol (25 mM), and 3 μM each of AroL, AroA, AroC, and PheA. The bottle was capped with a rubber stopper, through which two injection needles were inserted. The mixture of hydrogen and carbon dioxide was passed over the surface of the solution through the longer needle for 1 min at 4 °C. After incubation at 37 °C for 30 min, phenylpyruvic acid was determined as described above.

Part two determined the enzymatic activity of TyrB catalyzing the conversion of phenylalanine to phenylpyruvate acid^19^. The reaction system was as follows: 40 mM sodium cacodylate (pH 7.6), 80 μM pyridoxal phosphate and 2.5 mM phenylalanine, 5.0 mM 2-oxoglutarate, and TyrB at an appropriate concentration (2–5 μM). After incubation of mixtures at 37 °C for 30 min, the reaction was terminated by the addition of 0.2 ml of 1 M formic acid, and phenylpyruvic acid was determined as described above.

The activity of the crude enzyme extract was assayed by detection of phenylalanine. The reaction systems were as follows: Tris-HCl 100 mM, pH 7.5, SHIK (8 mM), ATP (8 mM), KCl (50 mM), MgCl_2_ (5 mM), PEP (4 mM), FAD (1 mM), NADH (5 mM), β-mercaptoethanol (25 mM), L-glutamic acid (4 mM), pyridoxal phosphate (1 mM) and appropriate crude enzyme extraction. The reaction was conducted at 37 °C for 30 min. The enzymes were inactivated by heating at 100 °C for ten minutes when the reaction was complete. The enzymes were then precipitated by centrifugation at 10,000 g for 5 min and the supernatant was used for detection of phenylalanine by HPLC, based on amino acid analysis with OPA derivatization and detection at 338 nm using Zorbax Eclipse-AAA columns on an Agilent (Santa Clara, USA) 1100 HPLC. The mobile phase consisted of two elutions: A (40 mM Na_2_HPO_4_, pH 7.8), B (ACN: MeOH: water = 45:45:10, v/v/v). The elution gradient profile was as follows: 0–1 min, 100% A; 9.8 min: 43% A + 57% B; 10 min: 100% B; 12 min: 100% B; 12.5 min: 100% A. The retention time of L-Phe was 26 min.

### Validation of the key enzyme in shikimate pathway *in vivo*

For the validation of the key enzyme identified in the multiple enzyme system *in vivo*, *aroL* and *aroA* genes were cloned into p15A1 to construct plasmids p15A1-aroL1/2/3 and p15A1-aroA1/2/3, respectively, using primers *aroL(A)*-LF11/12/13 and *aroL(A)*-LR11/12/13 (shown in Supplementary Table 1). *AroC* gene was cloned into p15A1 to construct plasmids p15A1-aroC1/2/3, used as controls. All the integration vectors (Table 1) used in this study were constructed by a sequence-independent “simple cloning” method without the need for restriction and ligation enzymes^32^. The host strains *HD-1* containing the different overexpression plasmids were fermented, separately, and the strain *HD-1* containing plasmid p15A1-aroA2 was fermented in a 5L-bioreactor.

## Results and Discussion

### L-phenylalanine-overproducing strain

In this study, *E. coli HD-1* strain was chosen as a host strain. It was derived from E. coli w3110 strain, which initially undergone multiple random mutagenesis and then overexpressing *aroF*^WT^ and *pheA*^fbr^ based on system-wide analysis of metabolism results in gradual increase in L-phenylalanine production throughout the strain engineering steps^27^. The genome sequence of the *HD-1* strain was determined and subjected to comparative genomic analyses (Supplementary Tables 2–3). With the genomic information of the w3110 strain as a control, pairwise genome alignment for the w3110 and *HD-1* strains revealed that a total 47 genes in the w3110 strains appeared to be either deleted or have modified sequences in the *HD-1*strain, the most effects of which on the L-phenylalanine production phenotype are not clear. Four genes involved in the L-phenylalanine biosynthesis of the *HD-1* strain, including *pykA*, *aroG*, *pta* and *pheA* genes, were found to have nonsynonymous SNPs, in comparison with the *E. coli* w3110 strain, which have likely affected L-phenylalanine production in the *HD-1* strain. Relatively genomic differences between *E. coli* w3110 and *HD-1*, and mutations in many of these L-phenylalanine biosynthesis-related genes indicate that the *HD-1* strain seems to be better optimized for the L-phenylalanine overproduction.

### Enzymatic assays for enzymes in the shikimate pathway

**Figure 1:**
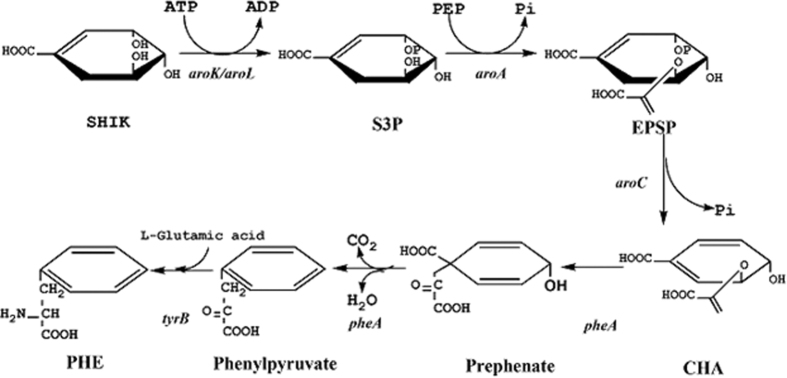
Shikimate acid pathway in *E. coli*. The reactions are catalyzed by shikimate kinase (AroK, AroL), EPSP synthase (AroA), chorismate synthetase (AroC), bifunctional chorismate mutase/prephenate dehydratase (PheA) and aromatic amino acid aminotransferase (TyrB).

**Figure 2:**
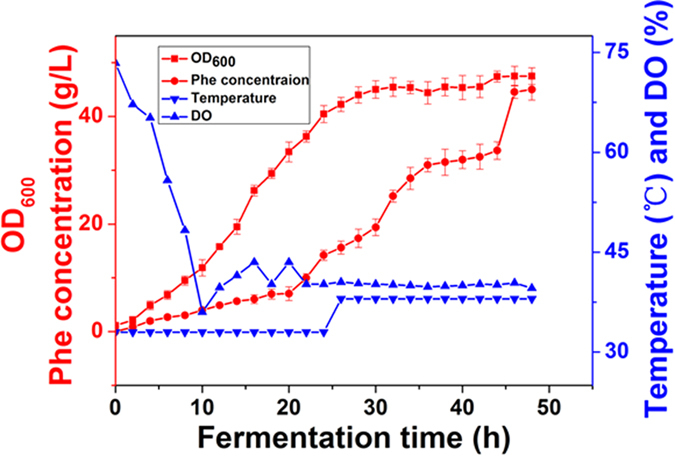
Fermentation process of phenylalanine production stain *HD-1*. The cells sampled at 32 h were used for proteomics analysis and crude enzyme extract experiments. The data is from three experimental replicates.

**Figure 3:**
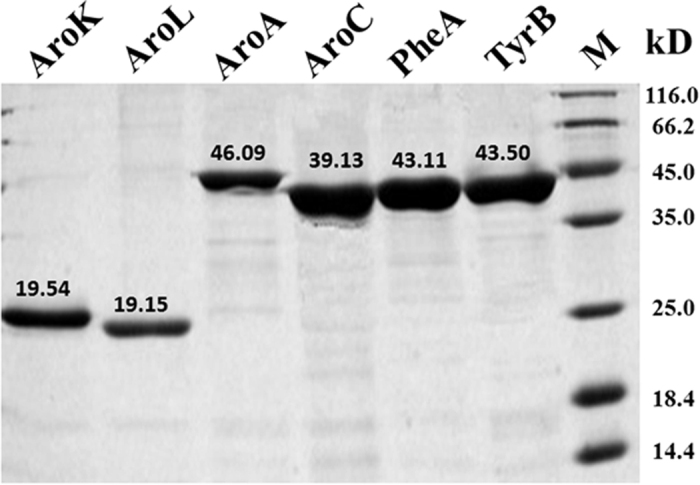
SDS-PAGE of the purified phenylalanine synthesis enzymes. Lane M, protein marker; lane 1, AroK; lane 2, AroL; lane 3, AroA; lane 4, AroC; lane 5, PheA; lane 6, TyrB.

**Figure 4:**
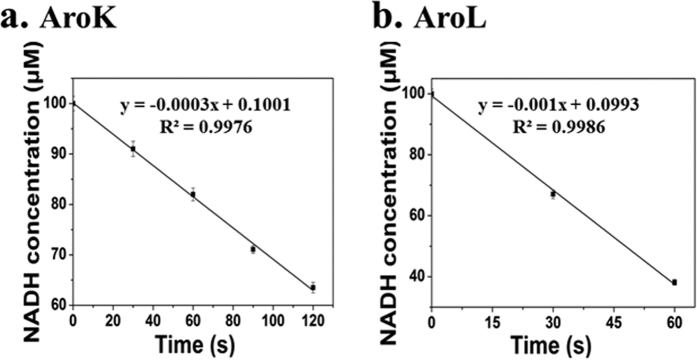
Determination of enzyme activity of AroK and AroL *in vitro*. (**a**) The enzyme activity of AroK; (**b**) The enzyme activity of AroL. The activities of the purified shikimate kinase was determined by coupling the release of ADP from the shikimate kinase-catalyzed reaction to the oxidation of NADH using pyruvate kinase and lactate dehydrogenase as coupling enzymes. Shikimate-dependent oxidation of NADH was monitored at *A*_340_ (ε = 6,200 M^−1^cm^−1^) at 25 °C. The data are from three experimental replicates.

**Figure 5:**
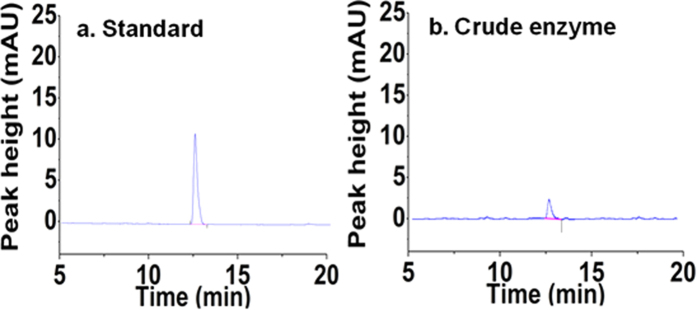
HPLC analysis of phenylalanine. (**a**) The peak from the standard metabolite of phenylalanine; (**b**) Phenylalanine produced in the *in vitro* reaction system constituted by crude enzyme extracts. The data are from three experimental replicates.

The five-step metabolic pathway for phenylalanine synthesis from SHIK in *E. coli* is illustrated in Fig. 1. To investigate the contribution of different enzymes to the production of L-Phe, the crude enzyme extract obtained from the cells at 32 h (Fig. 2) was used. The enzymes involved in the phenylalanine synthesis pathway were expressed in *E. coli* (DE3) and purified. The SDS-PAGE results from the *E. coli* express system are shown in Fig. 3. Specifically, the activities of two isoenzymes, namely AroK and AroL, were determined. The specific activity of AroK was 18 μmol/(min.mg), which was much lower than that of AroL (61 μmol/(min.mg), as shown in Fig. 4. This result indicated that AroL has a much better catalytic ability than AroK and may be the dominant enzyme of the pathway *in vitro*. The activities of the first four enzymes (AroL, AroA, AroC, and PheA) and the last one (TyrB) were quantitated by assaying phenylpyruvic acid production. The results revealed that all the purified enzymes were active, as shown in Table 2. Additionally, we measured the activities of the crude enzyme extract and detected a peak that appeared at relatively the same time as the phenylalanine standard (Fig. 5), indicating that all enzymes of the shikimate pathway present in the crude enzyme extract were functional and active.

### Determine the absolute enzyme concentrations

**Figure 6:**
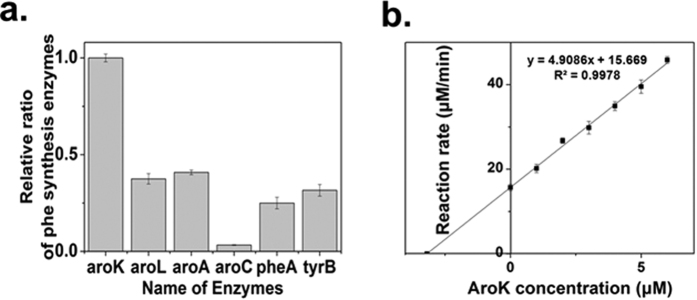
Determination of the absolute enzyme concentrations. (**a**) Relative ratios of the enzymes in the shikimate acid pathway. Original proteomics data was processed by APEX method. The relative amounts of enzymes in shikimate acid pathway were normalized to AroK. The data are from three experimental replicates. (**b)** The determination of AroK absolute concentration in the crude enzyme extract. Different amounts of purified AroK were added into the diluted crude extract and the reaction rates were determined. The AroK concentration in the diluted crude extract was calculated through the intersection with X-axis and the absolute concentration of AroK in the crude enzyme extract was calculated through the dilution rate. The data are from three experimental replicates.

Mass spectrometry data were processed using the Pioplite software with protein identification and quantification. The number of fragment-iron spectra acquired for all the peptides of a protein correlates with the expression level of each protein. However, as the differences in composition and size of each protein can be profound, these results are inadequate to accurately represent the expression level of different proteins. To overcome this problem, we used APEX^33^ to process the original label-free proteomics data to acquire reliable data on the ratios between different proteins. The relative amounts of enzymes (normalized to AroK) are shown in Fig. 6a. To determine the absolute intracellular concentrations of the various enzymes, at least one enzyme must be known in advance. We used AroK as such a standard, because the amount of AroK in the crude extract was easily measured by fluorescence since NADH is involved in its catalyzed reaction. Different amounts of purified AroK were added into the diluted (1:3) crude extract and the reaction rate was measured. The results revealed that the concentration of AroK in the crude enzyme extract was 9.6 μM, as can be seen from Fig. 6b. Combining the relative concentrations of the various intracellular enzymes, as determined by proteomics, and the AroK absolute concentration, the absolute concentrations of all the enzymes were calculated as follows: AroK 9.6 μM, AroL 3.67 μM, AroA 3.89 μM, AroC 0.32 μM, PheA 2.29 μM, TyrB 2.97 μM.

### Key enzymes in the phenylalanine synthesis pathway

**Figure 7:**
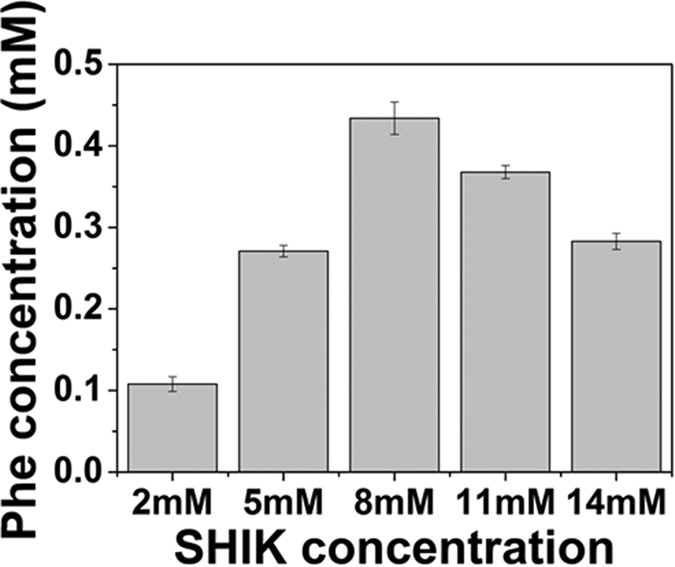
Influence of different substrate concentration on the production of Phe concentration *in vitro*. In this assay, SHIK was used as the substrate. The productions of phenylalanine were increased to different levels after adding different amounts of SHIK. The data is from three experimental replicates.

**Figure 8:**
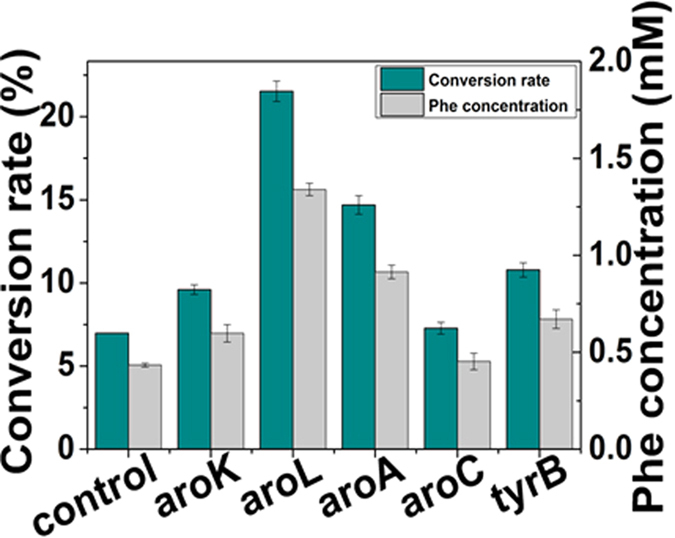
Reconstitution of enzyme concentrations in the *in vitro* system for phenylalanine productions. The yields of phenylalanine were increased to different levels after the addition of single purified enzymes to 2.5 times the level from the absolute enzyme concentrations measured in the crude cell extract, indicating the different impact of enzymes on the production of phenylalanine. The control is the production of phenylalanine in the system constituted by crude enzyme extract. The data is from three experimental replicates.

Since the concentrations of absolute enzyme in the crude extract were determined and the purified enzymes were available, we were able to measure the output of phenylalanine *in vitro* by adding purified enzymes into the crude extract. The metabolites added to the reaction system include: SHIK (8 mM), ATP (8 mM), KCl (50 mM), MgCl_2_ (5 mM), PEP (4 mM), FAD (1 mM), NADH (5 mM), β-mercaptoethanol (25 mM), L-glutamic acid (4 mM), and pyridoxal phosphate (1 mM). To ensure that the reaction rate was not affected by the low substrate concentration during the reaction, all the substrates were added at concentrations much higher than that of their Km values (Fig. 7). In order to evaluate the influence of different enzymes, each purified enzyme was separately titrated into the reaction system and the concentration of the corresponding enzymes was then increased to 2.5 times based on their initial level. After the addition of the purified enzymes, yields of phenylalanine were increased to different levels, indicating the different impact of each enzyme on the production of phenylalanine. The reaction system without adding any purified enzymes was used as control, as shown in Fig. 8. The production of phenylalanine in the samples in which AroL and AroA were added, was higher than in the other three groups, indicating that the two steps of the shikimate acid pathway (catalyzed by AroL and AroA, respectively) were potentially the limiting steps in the synthesis of phenylalanine. Thus, we reasoned that the genes *aroL* and *aroA* might be the bottlenecks causing the inefficiency in the production of phenylalanine.

### Validation of the key enzyme *in vivo*

**Figure 9:**
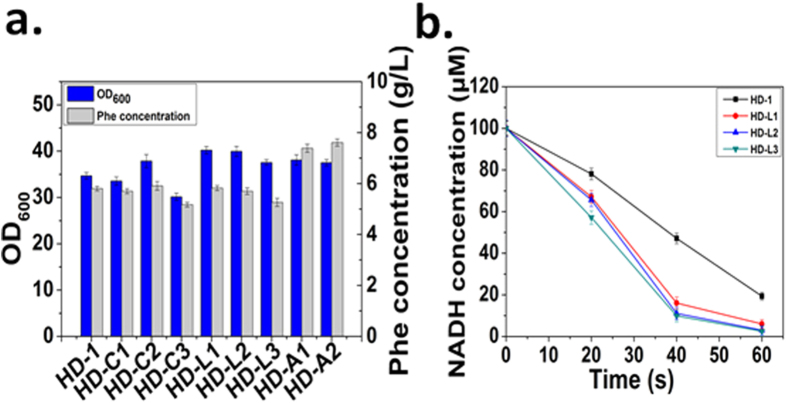
Fermentation results of the original *HD-1* strain and the *aroC*/*aroL/aroA* with different promoters overexpressed strain *HD-C1/2/3*, *HD-L1/2/3* and *HD-A1/2* in flask cultures. (**a**) The productions of phenylalanine when *aroC*, *aroL* and *aroA* were overexpressed respectively. The controls are the *HD-1* and *HD-C1/2/3* strains. (**b**) The enzyme activities of AroL in the strains *HD-L1/2/3* was determined by coupling the release of ADP from the shikimate kinase-catalyzed reaction to the oxidation of NADH using pyruvate kinase and lactate dehydrogenase as coupling enzymes. The control is the *HD-1* strain. The data is from three experimental replicates.

**Figure 10:**
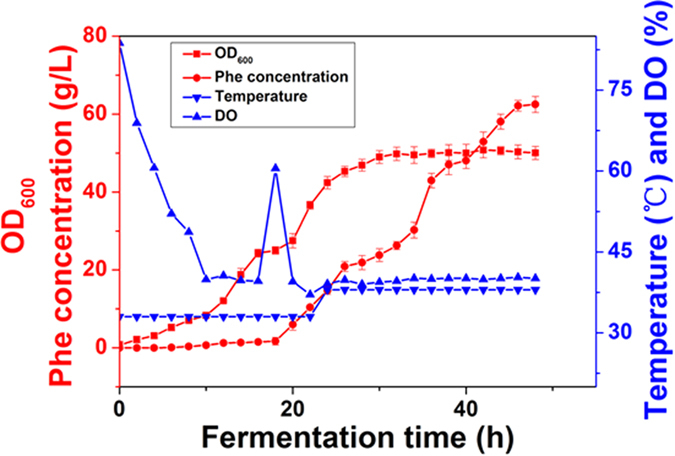
Fermentation process of phenylalanine production stain *HD-A2*. L-Phe production by *HD-A2* was investigated in a 5L-bioreactor, and the titer of phenylalanine was measured after 48 h cultivation. The data is from three experimental replicates.

The metabolic relevance of our observation *in vitro* was tested through overexpression of targeted genes *in vivo*. Nine strains (*HD-L (A, C)1/2/3*) carrying plasmids containing *aroL*, *aroA* or *aroC* under the control of different promoters were constructed. When we transformed the *HD-1* strain with p15A1-aroA3, which expresses the *aroA* gene under the strong promoter BBa_J23118, cell growth was severely reduced, likely due to overflow metabolism. As expected, as shown in Fig. 9a, the strains *HD-C1/2/3* overexpressing *aroC* did not improve the production of phenylalanine compared with *HD-1*. Unexpectedly, strains overexpressing *aroL*, *HD-L1/2/3*, did not lead to increased phenylalanine production. The *HD-L2/3* strains in particular produced 5.7 ± 0.13 g/L and 5.262 ± 0.16 g/L phenylalanine, which was slightly lower than the control strain *HD-1*. We hypothesized that this is mainly due to the inhibition of AroL by shikimate acid and tryptophan, which are not produced *in vitro*^4^,^34^,^35^. For example, the activity of AroL decreased by approximately sevenfold when the shikimate concentration was increased from 1 to 10 mM, suggesting an inhibition by high levels of the substrate^4^. Interestingly, the production of the other two resulting strains, *HD-A1/2,* reached 7.542 ± 0.16 g/l and, approximately 27% and 32% higher than the control strain *HD-1*, respectively. Actually, in order to ensure that AroL had been successfully overexpressed in the strains *HD-L1/2/3*, the enzyme AroL was purified and the activity of it was determined by coupling the release of ADP from the shikimate kinase-catalyzed reaction to the oxidation of NADH using pyruvate kinase and lactate dehydrogenase as coupling enzymes. As shown in Fig. 9b, the catalytic rate of the enzyme in strains *HD-L1/2/3* was significantly higher than that of the original strain *HD-1*. The result showed that the amount of AroL in the strains *HD-L1/2/3* was increased after the overexpression of the enzyme. At the same time, it was proved that AroL was successfully overexpressed in the strains *HD-L1/2/3*. The production of L-Phe by *HD-A2* and *HD-1* (the control) was also investigated in a 5 L-bioreactor, and the titer of phenylalanine reached 62.47 g/l after 48 h cultivation (Fig. 10). The engineered strain *HD-A2* produced approximately 38.82% more phenylalanine compared with the original strain *HD-1*, and the phenylalanine yield on glucose was increased from 0.186 g/g (18.6%) to 0.236 g/g (23.62%) (Table 3). EPSP synthase (AroA) catalyzes the transfer of the enolpyruvoyl moiety from phosphoenolpyruvate (PEP) to the hydroxyl group of carbon 5 of shikimate 3-phosphate. Therefore, we hypothesize that when the amount of AroA increases, it can improve the binding rate of PEP, thereby improving the reaction efficiency. This demonstrated that increasing expression of AroA, the key enzyme identified through *in vitro* metabolic control analysis, increased phenylalanine production *in vivo*.

## Conclusion

In this study, we have demonstrated that a metabolic pathway can be reconstituted *in vitro* from purified enzymes with cofactors and coenzymes, which is an ideal module to investigate its regulation since many parameters can be varied. The precise measurement of the absolute intracellular concentration of the enzymes *in vivo* and the possibility of fine-tuning of enzyme amounts *in vitro* make it possible to quantitatively evaluate the effect of different enzymes on the production of a specific pathway based on metabolic control analysis. In addition, key enzymes in the pathway can be determined and the overexpression of these enzymes is more likely to be an effective modification strategy to improve the carbon flux into the synthetic pathway of the targeted metabolites *in vivo*. The enzyme AroA exhibited the largest impact on phenylalanine synthesis in the phenylalanine producing strain *HD-A2*. The production in the modified strain *HD-A2* resulted in an increase of 38.82% in the L-Phe titer compared with the production in the original strain. This method, based on the reconstitution of purified stable enzymes *in vitro*, has unique advantages over blindly modifying living microorganisms *in vivo*. These include easy access and control, high product yield, fast reaction rate, tolerance of toxic compounds and products, great engineering flexibility for *in vitro* assembly and shifting unfavorable reaction equilibrium. This approach may shed light on a rational strategy for engineering strains and can serve as a new biomanufacturing platform evolving from fundamental research tools. Furthermore, it has a wide potential application for the efficient construction of industrial strains.

## Additional Information

**How to cite this article**: Ding, D. *et al.* Improving the Production of L-Phenylalanine by Identifying Key Enzymes Through Multi-Enzyme Reaction System *in Vitro. Sci. Rep.* **6**, 32208; doi: 10.1038/srep32208 (2016).

## Supplementary Material

Supplementary Information

## Figures and Tables

**Table 1:** Strains and plasmids used in this study.

Strains and plasmids	Description	Source
*HD-1*	L-phenylalanine overproducing strain. It was derived from *E. coli* W3110 strain, which initially undergone multiple random mutagenesis and then overexpressing *aroF*^WT^ and *pheA*^fbr^. The genome sequences of the *HD-1* strain were determined and compared (Supplementary Tables 2–3). The genome of this strain was used as the template for amplification of phenylalanine synthesis genes	This study
*DH5a*	This strain was used for the clone of the plasmid.	Lab stock
*BL21 (DE3)*	This strain was used for enzyme protein expression.	Lab stock
*HD-C1*	HD-1 p15A1-aroC1::aroC	This study
*HD-C2*	HD-1 p15A1-aroC2::aroC	This study
*HD-C3*	HD-1 p15A1-aroC3::aroC	This study
*HD-L1*	HD-1 p15A1-aroL1::aroL	This study
*HD-L2*	HD-1 p15A1-aroL2::aroL	This study
*HD-L3*	HD-1 p15A1-aroL3::aroL	This study
*HD-A1*	HD-1 p15A1-aroA1::aroA	This study
*HD-A2*	HD-1 p15A1-aroA2::aroA	This study
p15A1	p15A derivative, carrying *pheA*^*f*br^ *aroF*^wt^ gene, Kan^r^	Lab stock
pET28-a(+)	*E. coli* expression plasmid, T7 promoter, T7 terminator, Kan^r^	Lab stock
pET28a-aroL	N-terminal His-tagged *aroL*, inserted between *Bam*HI and *Eco*RI sites of pET 28a (+)	This study
pET28a-aroK	N-terminal His-tagged *aroK*, inserted between *Bam*HI and *Eco*RI sites of pET 28a (+)	This study
pET28a-aroA	N-terminal His-tagged *aroA*, inserted between *Bam*HI and *Eco*RI sites of pET 28a (+)	This study
pET28a-aroC	N-terminal His-tagged *aroC*, inserted between *Eco*RI and *Hin*dIII sites of pET 28a (+)	This study
pET28a-pheA	N-terminal His-tagged *pheA*, inserted between *Bam*HI and *Eco*RI sites of pET 28a (+)	This study
pET28a -tyrB	N-terminal His-tagged *tyrB*, inserted between *Bam*HI and, *Eco*RI sites of pET 28a (+)	This study
p15A1-aroC1	p15A-aroF^wt^-pheA^fbr^ derivative, carrying *aroC* gene, BBa_J23116 promoter, Kan^r^	This study
p15A1-aroC2	p15A-aroF^wt^-pheA^fbr^ derivative, carrying *aroC* gene, BBa_J23107 promoter, Kan^r^	This study
p15A1-aroC3	p15A-aroF^wt^-pheA^fbr^ derivative, carrying *aroC* gene, BBa_J23118 promoter, Kan^r^	This study
p15A1-aroL1	p15A-aroF^wt^-pheA^fbr^ derivative, carrying *aroL* gene, BBa_J23116 promoter, Kan^r^	This study
p15A1-aroL2	p15A-aroF^wt^-pheA^fbr^ derivative, carrying *aroL* gene, BBa_J23107 promoter, Kan^r^	This study
p15A1-aroL3	p15A-aroF^wt^-pheA^fbr^ derivative, carrying *aroL* gene, BBa_J23118 promoter, Kan^r^	This study
p15A1-aroA1	p15A-aroF^wt^-pheA^fbr^ derivative, carrying *aroA* gene, BBa_J23116 promoter, Kan^r^	This study
p15A1-aroA2	p15A-aroF^wt^-pheA^fbr^ derivative, carrying *aroA* gene, BBa_J23107 promoter, Kan^r^	This study
p15A1-aroA3	p15A-aroF^wt^-pheA^fbr^ derivative, carrying *aroA* gene, BBa_J23118 promoter, Kan^r^	This study

Kan, kanamycin; r, resistance.

**Table 2:** The determination of purified enzyme activity.

	Enzyme fraction	Phenylpyruvate formed (μM)
Control	aroL, aroA	0
Part 1	aroL, aroA, aroC, pheA	0.26
Part 2	tyrB	0.32

Incubation mixture was described in the “Assay of the enzyme activities”. Phenylpyruvate was determined as described in the “Determination of phenylpuruvate acid”.

**Table 3:** Comparison of the fermentation results of the original *HD-1* strain and the *aroA* overexpressed strain *HD-A2* by fed-batch fermentation.

	OD_600_	Phe concentration (g/L)	Phe yield on glucose (%)
*HD-1*	47.49 ± 2.21	45 ± 1.79	18.6 ± 0.97
*HD-A2*	50.04 ± 1.97	62.47 ± 2.03	23.62 ± 1.04

The OD, Phe concentration and yield were compared between *HD-1* and *HD-A2*. The data is from three experimental replicates.
