# Supplementary material for: Improving the Production of L-Phenylalanine by Identifying Key Enzymes Through Multi-Enzyme Reaction System in Vitro
Source: Sci Rep. 2016 Aug 25;6:32208. doi: 10.1038/srep32208 (PMC4997321; doi:10.1038/srep32208)
Supplement: Supplementary Information [file srep32208-s1.docx]

**Improving the Production of L-Phenylalanine by Identifying Key Enzymes Through Multi-Enzyme Reaction System in Vitro**

Dongqin Ding^1,2^, Yongfei Liu^1,3^, Yiran Xu^1^, Ping Zheng^1,3^, Haixing Li^2^, Dawei Zhang^1,3*^ and Jibin Sun^1,3^

*Corresponding authors. Xi Qi Dao #32, Tianjin airport Economic area, Tianjin China. 300308. [zhang_dw@tib.cas.cn](mailto:zhang_dw@tib.cas.cn) (D. Zhang) +86-22-24828749

^1^ Tianjin Institute of Industrial Biotechnology, Chinese Academy of Sciences, Tianjin 300308, People’s Republic of China.

^2^ Department of Food Science and Engineering, School of Food, Nanchang University, Nanchang 330029, People’s Republic of China.

^3^ Key Laboratory of Systems Microbial Biotechnology, Chinese Academy of Sciences, Tianjin 300308, People’s Republic of China.

**Supplementary Table 1 PCR primers used in this study**

| Enzyme name | Prime name | Primer sequence（5'to3'） |
| --- | --- | --- |
| aroL | *aroL*_F1 | CGCGGATCCATGACACAACCTCTTTTTCT |
|  | *aroL*_R1 | CCGGAATTCTCAACAATTGATCGTCTGTG |
| aroK | *aroK*_F1 | CGCGGATCCATGGCAGAGAAACGCAATAT |
|  | *aroK_R1* | CCGGAATTCTTAGTTGCTTTCCAGCATGT |
| aroA | *aroA*_F1 | CGCGGATCCATGGAATCCCTGACGTTACA |
|  | *aroA*_R1 | CCGGAATTCTCAGGCTGCCTGGCTAATCC |
| aroC | *aroC*_F1 | CCGGAATTCATGGCTGGAAACACAATTGG |
|  | *aroC*_R1 | CCCAAGCTTTTATTACCAGCGTGGAATATCAG |
| pheA | *pheA*_F1 | CGCGGATCCATGACATCGGAAAACCCGTT |
|  | *pheA*_R1 | CCGGAATTCTCAGGTTGGATCAACAGGCA |
| tyrB | *tyrB*_F1 | CGCGGATCCGTGTTTCAAAAAGTTGACGC |
|  | *tyrB_*R1 | CCGGAATTCTTACATCACCGCAGCAAACG |
| aroC  aroC  aroC  aroL  aroL  aroL | *aroC_*F11  *aroC_*R11  *aroC_*F12  *aroC_*R12  *aroC_*F13  *aroC_*R13  *aroL_*F11  *aroL_*R11  *aroL_*F12  *aroL_*R12  *aroL_*F13  *aroL_*R13 | TTGACAGCTAGCTCAGTCCTAGGGACTATGCTAGCAACTTTAAGAAGGAGATATACATATGGCTGGAAACACAATTGGACAAC  TTACCAGCGTGGAATATCAGTCTTC  TTTACGGCTAGCTCAGCCCTAGGTATTATGCTAGCAACTTTAAGAAGGAGATATACATATGGCTGGAAACACAATTGGACAAC  TTACCAGCGTGGAATATCAGTCTTC  TTGACGGCTAGCTCAGTCCTAGGTATTGTGCTAGCAACTTTAAGAAGGAGATATACATATGGCTGGAAACACAATTGGACAAC  TTACCAGCGTGGAATATCAGTCTTC  TTGACAGCTAGCTCAGTCCTAGGGACTATGCTAGCAACTTTAAGAAGGAGATATACATATGACACAACCTCTTTTTCTGATCG  TCAACAATTGATCGTCTGTGCCAGG  TTTACGGCTAGCTCAGCCCTAGGTATTATGCTAGCAACTTTAAGAAGGAGATATACATATGACACAACCTCTTTTTCTGATCG  TCAACAATTGATCGTCTGTGCCAGG  TTGACGGCTAGCTCAGTCCTAGGTATTGTGCTAGCAACTTTAAGAAGGAGATATACATATGACACAACCTCTTTTTCTGATCG  TCAACAATTGATCGTCTGTGCCAGG |
| aroA  aroA  aroA | *aroA_*F11  *aroA_*R11  *aroA_*F12  *aroA_*R12  *aroA*_F13  *aroA_*R13 | TTGACAGCTAGCTCAGTCCTAGGGACTATGCTAGCAACTTTAAGAAGGAGATATACATATGGAATCCCTGACGTTACAACCCA  TCAGGCTGCCTGGCTAATCCGCGCC  TTTACGGCTAGCTCAGCCCTAGGTATTATGCTAGCAACTTTAAGAAGGAGATATACATATGGAATCCCTGACGTTACAACCCA  TCAGGCTGCCTGGCTAATCCGCGCC  TTGACGGCTAGCTCAGTCCTAGGTATTGTGCTAGCAACTTTAAGAAGGAGATATACATATGGAATCCCTGACGTTACAACCCA  TCAGGCTGCCTGGCTAATCCGCGCC |

**Supplementary Table 2 List of candidate SNPs found between genomes of the *E.coli* W3110 and *HD-1* strains**

| Candidate SNP patterns | Location in the genome of W3110 | Location in the genome of HD-1 | Gene name |
| --- | --- | --- | --- |
| G->A | 357606 | 357606 | cynR |
| C->T | 483957 | 483957 | acrA |
| C->T | 756646 | 756646 | sdhA |
| C->T | 786264 | 786264 | aroG |
| G->T | 987574 | 987574 | ompF |
| T->C | 1093686 | 1093686 | ycdT |
| T -> C | 1655771 | 1655771 | rspA |
| C->T | 1764125 | 1764125 | sufC |
| C->T | 1816268 | 1816268 | katE |
| G->A | 1837233 | 1837233 | ynjA |
| G->T | 1949953 | 1949953 | nudB |
| C->T | 2038532 | 2038532 | hchA |
| C->A | 2174089 | 2174089 | gatD |
| C->T | 2420398 | 2420398 | pta |
| G->A | 2607227 | 2607227 | hyfG |
| A->C | 2737376 | 2737376 | pheA |
| G->A | 2800287 | 2800287 | nrdE |
| G->A | 2805593 | 2805593 | proW |
| G->A | 2823497 | 2823497 | mltB |
| G->A | 2824550 | 2824550 | srlA |
| C->T | 3180977 | 3180977 | zupT |
| C->T | 3434264 | 3434264 | zraS |
| C->T | 3485433 | 3485433 | ppc |
| C->T | 3652233 | 3652233 | aslA |
| G->A | 3746911 | 3746911 | tnaB |
| C->T | 3815625 | 3815625 | trmH |
| C->T | 3843029 | 3843029 | rfaL |
| C->T | 3864854 | 3864854 | mltD |
| T ->C | 3868712 | 3868712 | yibI |
| C->T | 3890582 | 3890582 | sgbU |
| C->T | 3905935 | 3905935 | xylH |
| C->T | 4104141 | 4104141 | ompR |
| C->T | 4107008 | 4107008 | pck |
| G->A | 4134844 | 4134844 | yhfS |
| T -> C | 4153609 | 4153609 | yhfK |
| G->A | 4240443 | 4240443 | yjbF |
| G->A | 4245416 | 4245416 | xylE |
| G->A | 4246963 | 4246963 | malG |
| A ->G | 4251277 | 4251277 | malK |
| G->A | 4455778 | 4455778 | ytfR |
| G->A | 4507288 | 4507288 | insG |
| C->T | 4534692 | 4534692 | sgcC |
| C->A | 4628388 | 4628388 | lplA |

**Supplementary Table 3 List of genes absent in the genome of *HD-1***

| locus_tag | product | Gene name |
| --- | --- | --- |
| BAA16036 | transcriptional repressor for the insertion element IS2 | insC |
| BAA22515 | S2 element protein | insD |
| BAE77585 | the transposase for the insertion sequence element IS5 | insH |
| BAE77766 | proton motive force-dependent C4-dicarboxylate transporter | dctA |
